# Phototactic Algae-Driven Unidirectional Transport of Submillimeter-Sized Cargo in a Microchannel

**DOI:** 10.3390/mi10020130

**Published:** 2019-02-16

**Authors:** Moeto Nagai, Takahiro Hirano, Takayuki Shibata

**Affiliations:** Department of Mechanical Engineering, Toyohashi University of Technology, Toyohashi, Aichi 441-8580, Japan; saucy_cat1030@yahoo.co.jp (T.H.); shibata@me.tut.ac.jp (T.S.)

**Keywords:** microrobot, phototaxis, *Volvox*, micro-swimmer, biohybrid system, motion control

## Abstract

The sensing and actuation capabilities of biological cells integrated with artificial components have been used to create autonomous microsystems. For creating autonomous microsystems, the unidirectional transport of a submillimeter-sized cargo with stimuli responsive bio-motors should be developed as a fundamental motion. This study aims to use *Volvox* as a light-controlled microrobot to achieve the unidirectional transport of a submillimeter-sized cargo. We show the fabrication of a guide structure, cargo, and light irradiation platform for a unidirectional actuation. The fundamental performances of each component were investigated, and the motions of *Volvox* were controlled in a microchamber with the developed light irradiation platform. All components were integrated to demonstrate the unidirectional actuation of a block by *Volvox*. We discuss the dynamics of the mechanical motions.

## 1. Introduction

Controllable micro-robots are essential for tasks in the micro scale such as transportation, regulation, and assembly. Such micro-robots are still difficult to fabricate artificially using current microfabrication technologies, because these technologies rely on two-dimensional processes [1,2]. Artificial micro-/nanomotors have been produced from asymmetric spherical and tubular structures and recent studies demonstrated light-controlled movements [3,4,5] and taxis-behaviors [6,7]; nevertheless, they often require the precise regulation of an operating environment. Natural micro-robots in living organisms can stably work under a wider range of environmental conditions and are more sophisticated than artificial ones. These biological micro-robots perform multiple functions of motor, sensory receptor, and metabolism [8]. To take full advantage of their sensing and actuation capabilities, biological cells have been integrated with artificial components to create bio-hybrid systems. These investigations have led to the development of novel microsystems and the increased functionality of them. Cell actuators have powered autonomous bio-hybrid tweezers [9], mixers [10,11], gear [12], and pumps [13,14] in the microscale.

Biological motors are responsive to external signals, and the motions of living organisms have been controlled using magnetic [15,16,17,18,19], electrical [20,21,22,23], chemical [24,25,26,27], and optical [12,23,28,29,30,31,32,33,34] methods. Among them, the optical methods are advantageous in that cellular motions are controlled remotely without contacting the cells, which provides light-controlled or light-responsive systems. Light-sensitive motors are expected to be a micro-control unit with an embedded light sensor for autonomous microsystems.

Regardless of the motion control of biological motors, micrometer to millimeter objects have been transported by unicellular and multicellular micro-swimmers. Micrometer objects were transported by unicellular organisms such as *Escherichia coli* (~2 μm in length) [27,35,36,37], *Magnetococcus marinus* (1–2 μm in diameter) [18], *Serratia marcescens* (several micrometers in length) [23,30], bovine sperm (10 μm in length) [38], and *Chlamydomonas reinhardtii* (10 μm in diameter) [31]. To actuate an order of magnitude for a larger object or more than the size of unicellular micro-swimmers, their collective motion should be used [23,30,37]. Multicellular micro-swimmers spontaneously express the collective motion of somatic cells, and they are more convenient for actuating submillimeter to millimeter objects than unicellular micro-swimmers. Millimeter objects were driven by multicellular organism such as *Volvox* aureus (200–500 μm in diameter) [32] and *Artemia salina* (~several mm in length) [33,34]. Specifically, a gear was floated without a shaft, and it was rotated and translated by *V. aureus* [32] and *A. salina* [33]. *A. salina* moved a millimeter float along a guide [34]. The unidirectional transport of a submillimeter-sized cargo without rotation is a fundamental mechanism and in demand for applications in autonomous microsystems, yet it has not been achieved.

Among micro-swimmers, *Volvox carteri* is suitable for actuating sub-mm objects, because *Volvox* shows positive phototaxis and swims by means of flagella on thousands of surface somatic cells [39]. Moreover, *V. carteri* has a diameter of approximately 200 μm [40] and its total swimming force is ~1 nN [41]. Since a pusher-type swimmer creates a flow field away from the cell [42], nearby pushers can transport cargo. On the other hand, the velocity field around *V. carteri* shows it is a neutral swimmer [43,44]. We hypothesize that *V. carteri* should be adjacent to the wall of a block to actuate a cargo in the swimming direction. The objective of this study is to use *Volvox* as a light-controlled microrobot and achieve the unidirectional transport of a submillimeter-sized cargo. This achievement requires the combination use of *Volvox*, a guide structure, cargo, and a light irradiation platform. In this paper, we prepare a guide structure, cargo, and light irradiation platform for a unidirectional actuation by *Volvox.* We investigate the fundamental performance of each component. All components are integrated to demonstrate the unidirectional actuation by *Volvox* and the mechanical motions are characterized.

## 2. Materials and Methods

### 2.1. Overview of Experimental Setup

**Figure 1:**
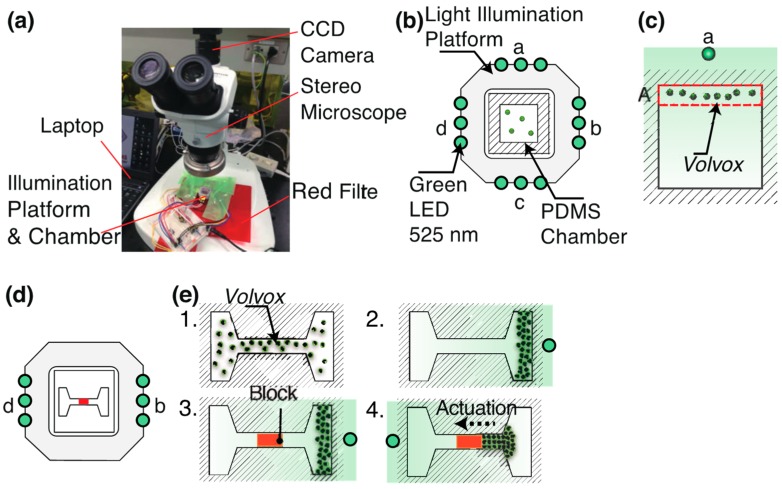
Experimental methods for the demonstration of (**b**), (**c**) phototactic control and (**d**), (**e**) unidirectional transport. (**a**) Picture of an experimental setup. (**b**), (**d**) schematic of the experimental setup. (**c**) Phototactic migration of *Volvox* to region A with LED *a*. (**e**) Experimental processes for the actuation of a movable block by *V. carteri*.

Figure 1 shows the experimental setup used in this study. We developed a common light irradiation platform and set two types of micro chambers where *V. carteri* and a microblock were introduced. This platform allowed light irradiation from four directions by using LEDs of a, b, c, and d. We selected a green LED (emission maximum centered at λ = 525 nm, outer diameter: 3 mm, Stanley Electric, UG3803X, Tokyo, Japan) for controlling the motion of *V. carteri*. LED light was illuminated from one side to induce the migration of *V. carteri*.

The colonies have positive phototaxis and move toward light sources [45]. *V. carteri* (NIES-865) was obtained from the Microbial Culture Collection at the National Institute for Environmental Studies (NIES), Japan and cultured in either a MG medium (NIES) or algae universal medium (Wamushiya). *V. carteri* was illuminated by a white LED light in a daily cycle of 14 h of light and 10 h of darkness. The intensity of the growth light was 4000 lux.

A microchip was mounted on the stage of a stereo microscope (Olympus, SZ61, Tokyo, Japan). *V. carteri* was placed into the chamber and observed under the stereo microscope equipped with a camera (Point Grey Research, Grasshopper Express, Richmond, BC, Canada). The illumination light of the microscope was filtered with a red transmission filter to prevent the photo responses to the bright-field illumination from the halogen lamp. The motion of the colonies was analyzed using image analysis software (ImageJ, Version 1.52).

### 2.2. Phototaxis and Swimming Speed Evaluation of Volvox carteri in Square Microchamber

We controlled the motions of *V. carteri* by switching the LEDs approximately every 70 s (Figure 1b,c). The chamber was divided into four regions, A, B, C, and D. The number of colonies in the regions was counted every 5 s to evaluate their phototaxis. We also measured each swimming distance, Δ*d* in time, Δ*t*. Δ*d*/Δ*t* gives the swimming speeds of *Volvox* colonies. 100 *Volvox* colonies were randomly selected from a recorded video. We plotted a distribution diagram of the swimming speeds of *V. carteri.*

We designed a square microchamber (vertical 9.4 mm × horizontal 9.4 mm × height 0.65 mm) in AutoCAD and fabricated it in polydimethylsiloxane (PDMS, Silpot 184, Dow Corning Toray, Tokyo, Japan) by softlithography. PDMS base and curing agents were mixed in a weight ratio of 10:1. The detailed fabrication process is described in the Supplementary Materials. Briefly, a PDMS chamber was replicated from a printing plate (relief depth 0.65 mm, Fujifilm, Torelief DWF95DTN, Toray Industries, Tokyo, Japan) and bonded to a glass substrate to form a closed PDMS chamber.

### 2.3. Unidirectional Transport of the Block by Volvox

Figure 1d,e show the experimental setup and procedures for transporting a block. Colonies of *V. carteri* and a movable block were placed in a PDMS chamber. The motions of *Volvox* colonies were controlled by switching between LED *b* and LED *d* on both sides of the platform to control their movements. Their driving forces transported the block unidirectionally. 

We prepared this setup via the following steps: (1) An open top PDMS and block were fabricated according to the methods described in Section 2.4. They were immersed in a solution of surfactants (Pluronic^®^ F-127, Sigma-Aldrich, St. Louis, MO, USA) to prevent the suction of the block to the PDMS channel. (2) The suspension of *V. carteri* in a culture medium was transferred to the chamber with a syringe. (3) The motion of the *Volvox* colonies was controlled by turning on LED *b* or *d* to migrate them to one side of the chamber. (4) While *V. carteri* colonies were trapped, a block was placed manually in the center of the channel with tweezers. (5) *Volvox* colonies were steered to the other side with LED *b* or *d* to actuate the block. (6) The block continued to experience the collective swimming motion of *Volvox* colonies and its motion was observed with a stereo microscope. The position of the block structure was recorded every 10 s. The average driving speed of the block was measured from an actuation distance and time.

### 2.4. Fabrication of Thick SU-8 Structure with a Rubber Ring

**Figure 2:**
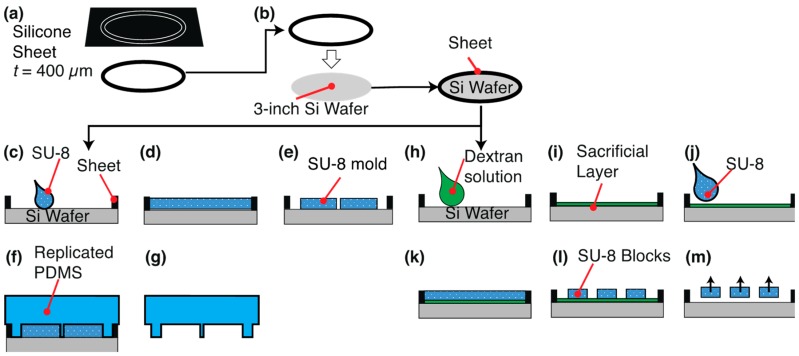
Schematic of a SU-8 mold fabrication process. (**a**) Ring formation from a silicone sheet. (**b**) Placement of a rubber sheet on a wafer. (**c**–**g**) Fabrication of a polydimethylsiloxane (PDMS) microchamber from a SU-8 mold. (**c**) SU-8 dispensing, (**d**) flattening by gravity. (**e**) SU-8 photolithography. (**f**) PDMS molding. (**g**) Release of the PDMS micro chamber. (**h**–**m**) Fabrication of the SU-8 blocks. (**h**) Dispensing of a dextran solution. (**i**) Spin-coating of the dextran. (**j**) SU-8 dispensing. (**k**) Flattening by gravity. (**l**) SU-8 photolithography. (**m**) Release of the SU-8 blocks in water.

We fabricated a submillimeter-sized thick SU-8 structure with a silicone rubber ring, which had a function of storing a thick SU-8 resist. Our method was based on SU-8 casting [46]. A rubber sheet with a thickness of 0.4 mm was patterned with a cutting machine (silhouette CAMEO, GRAPHTEC Corp., Yokohama, Japan) to fit a 3-inch diameter with a 2 mm width (Figure 2a). The rubber sheet was pasted in a wafer (Figure 2b).

**Figure 3:**
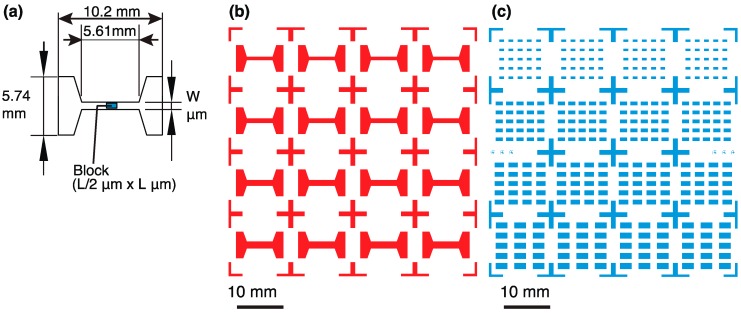
Design of a block and chamber for the actuation experiment by *Volvox*. (**a**) Dimensional drawing of a single block integrated in a chamber. Photomask patterns of (**b**) chambers and (**c**) blocks.

We designed a photomask of a micro chamber and block, which were required for the unidirectional transportation of a block by *V. carteri* (Figure 3). One combination of a block and chamber is shown in Figure 3a. All patterns of the chambers and blocks are displayed in Figure 3b,c. We used SU-8 3050 (12,000 cSt, 75.5% solids, Microchem, Newton, MA, USA) and calculated a required volume of SU-8. The original solution of SU-8 was diluted with cyclopentanone at a weight ratio of 1:1 to decrease its viscosity. This diluted SU-8 was poured onto a wafer with a ring and spread uniformly.

The photolithography of a thick SU-8 resist was processed under the common following conditions. (1) SU-8 was prebaked on a hot plate to cure the coated resist at 65 °C (15 min), 95 °C (240 min), and 65 °C (15 min). (2) We used a double-sided aligner (PEM-800, Union Optical, Tokyo, Japan) and the wafer was exposed to reach a light integral of 2500 mJ/cm^2^ through a polymethylmethacrylate (PMMA) filter (3) The resist was post-baked at 65 °C (6 min), 95 °C (15 min), and 65 °C (6 min). (4) The SU-8 coated substrate was developed in 2-methoxy-1-methylethyl acetate for 15 min. The SU-8 was rinsed in acetone and blown by a nitrogen gas.

### 2.5. Fabrication of Open-Top PDMS Chamber and SU-8 Blocks

A photomask of single micro chambers is shown in Figure 3b. One chamber is 5.5 mm in width and 10.2 mm in length. A flow channel with a length of about 5.6 mm was created for the rectified actuation of a block in the center. The sizes of the blocks were designed to be 500 μm × 1000 μm, 750 μm × 1500 μm, 1000 μm × 2000 μm, and 1250 μm × 2500 μm (Width × Length). To actuate these blocks, the gap between the block and a guide channel was set to 100 μm. Therefore, we prepared four types of channel widths, 0.7 mm, 0.95 mm, 1.2 mm, and 1.45 mm.

An open top PDMS chamber was fabricated using the following methods. (1) We weighed 2.5 g of SU-8 3050 to make a 300 μm height mold and diluted SU-8 was poured on a silicon wafer (Figure 2c). (2) The resist was flattened by gravity (Figure 2d). (3) Photolithography (Figure 2e). (4) A 10 μL of Trichloro(1H,1H,2H,2H-perfluorooctyl)silane was vaporized and exposed to the SU-8 mold for ease of releasing. (5) PDMS (10:1 base:curing agent) mixture was poured over the SU-8 mold and air bubbles were removed in a vacuum chamber. (6) The PDMS was cured at 80 °C for 2 h in an oven (Figure 2f). (7) The cured PDMS was peeled off from the mold (Figure 2g).

250 μm thick blocks were fabricated similarly to the microchamber by photolithography. The major difference is the spin-coating of a sacrificial layer on a silicon wafer before the resist coating. (1) 10% (w/v) aqueous solution of dextran was spin-coated at 1000 rpm for 20 s (Figure 2h). We selected a 10% (w/v) dextran (Mw: 60,000) as a sacrificial layer. (2) A dextran film was formed on a hot plate at 150 °C for 2 min (Figure 2i). (3) We weighed 2.1 g of SU-8 3050 for the structure and diluted SU-8 was poured on the wafer (Figure 2j). (4) Photolithography (Figure 2k,l). (5) Release of the blocks in water (Figure 2m). 

The dimensions of the structures were observed with a microscope (VHX-2000, KEYENCE, Osaka, Japan). The height of the chambers and blocks was measured by observing the cross-sections. 

## 3. Results and Discussion

### 3.1. Phototactic Steering of V. carteri in a Square Chamber

**Figure 4:**
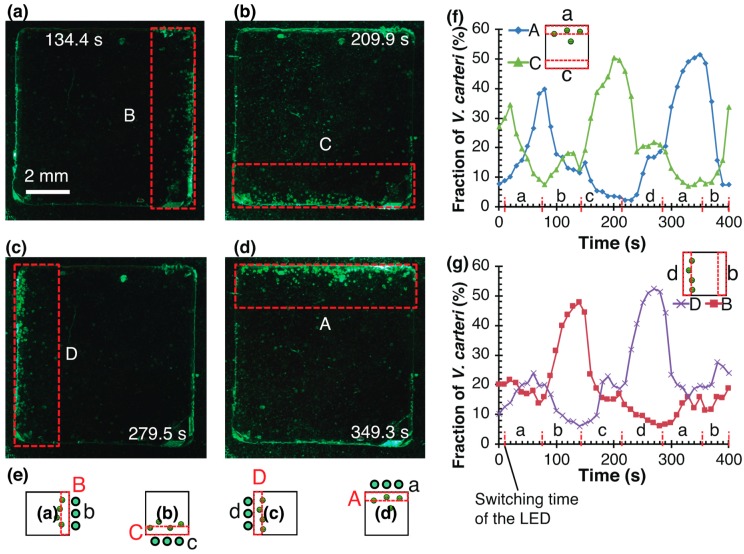
Motion control results of *V. carteri* in a closed micro chamber with the light irradiation platform. (**a**–**d**) Micrographs showing a state of motion control of *V. carteri* (green points). (**e**) Schematic of *Volvox* in areas A–D with LEDs a–d. (**f**), (**g**) time is plotted on the horizontal axis and the number of *V. carteri* is plotted on the vertical axis. These graphs separately plot A, C and B, D that face each other. Red dashed lines represent the switching time of the LED (*a*, *b*, *c*, *d*).

Colonies of *V. carteri* were injected into a closed square chamber and migrated to a light irradiation direction. They followed the changes of the light directions switched approximately every 70 s (Figure 4). After reaching the edge near the lit LED, *Volvox* stayed in their places. Figure 4a–d show migrated *Volvox* colonies in four regions, A, B, C, and D, where the time stamps in the figure correspond to the peak number in each condition. Figure 4e illustrates four conditions of *Volvox* in areas A–D with LEDs a–d. Figure 4f,g plot the number of *Volvox* colonies in the four regions taken at every five seconds. Light illumination increased the number of *Volvox* in the area closest to the lit LED and decreased the number in the opposite region. Approximately 50% of the total 214 colonies moved to the following region within about 70 s. A typical distance from one region to the next region is 7 mm. The speeds obtained from the time and travelling distance agreed with the swimming speeds of *Volvox* ranging 0 μm/s to about 350 μm/s [44]. The motion control experiments indicate the collective motion can be used for the transportation of a block. The number of *Volvox* colonies 214 and the volume of a chamber 57 mm^3^ (=9.4 × mm × 9.4 mm × 0.65 mm) give the density of *Volvox*, 4 colonies/mm^3^.

**Figure 5:**
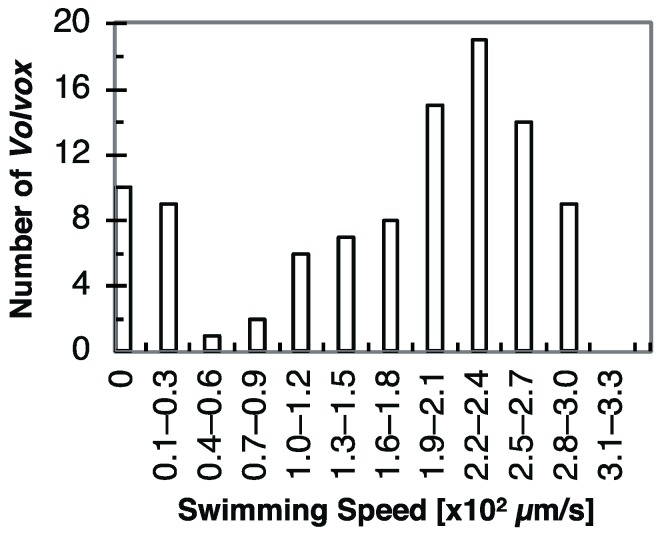
Swimming speed distribution of *Volvox carteri* calculated by the equation in Section 2.2. The horizontal axis is the swimming speeds and the vertical axis is the frequency of the population.

We acquired swimming videos and evaluated the swimming speeds of 100 randomly selected *Volvox* colonies in a micro chamber. Figure 5 shows a histogram of a swimming speed distribution of *V. carteri*, which was calculated from each swimming distance and time. This distribution is bimodal and has two peaks at around 0 μm/s and 230 μm/s. The average is approximately 170 μm/s and the mode is 230 μm/s. The left peak can be explained by the rest of the horizontal movement of about 20% of the total *Volvox,* which remained at the same places. They were stuck or their motilities were naturally lower. This stoppage was most likely caused by the trapping of the *Volvox* between the PDMS and the glass substrates.

### 3.2. Fabrication of Thick Open-Top Chambers and SU-8 Blocks

**Figure 6:**
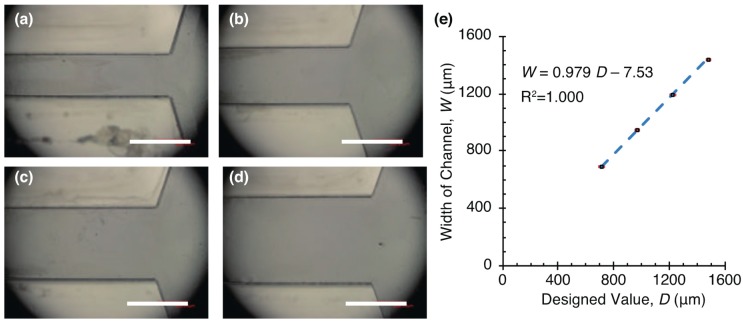
Fabrication of thick SU-8 molds for an open-top microchamber. Microscope image of channels with widths of (**a**) 714 μm, (**b**) 969 μm, (**c**) 1225 μm, and (**d**) 1480 μm. Scale bars: 1 mm. (**e**) Actual widths of SU-8 channels versus designed values.

An open-top PDMS chamber and SU-8 blocks were fabricated with diluted SU-8 and a rubber ring. Thick SU-8 molds for a chamber were fabricated (Figure 6) and PDMS chambers were replicated from the molds. We measured the widths and thickness of the channels and plotted the width of channels in Figure 6e. The measurement results show that the mold was fabricated with a shrinkage of about 2–3% from the designed value. The PDMS chamber was cut with a razor and we measured the chamber height by observing the cross section with the microscope. We obtained a uniform height chamber with a height of 238.9 ± 22.1 μm (mean ± standard deviation, n = 35 points). Dilution of the resist lowered the viscosity of SU-8 and enabled uniform thickness of the chamber. 

**Figure 7:**
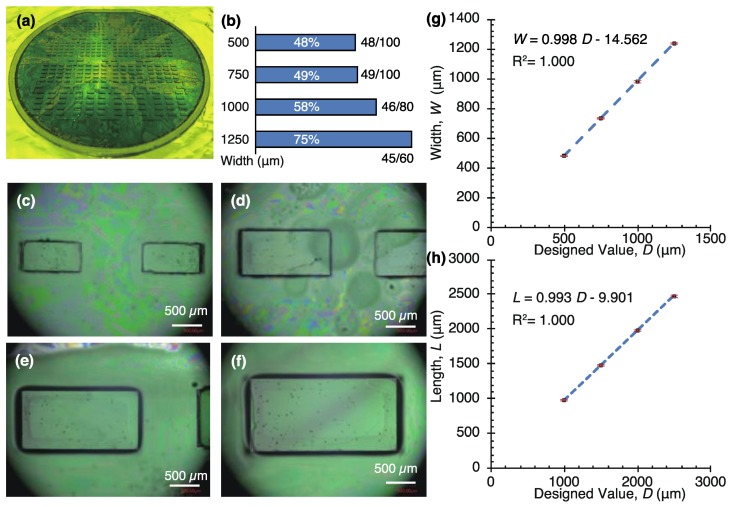
Fabrication results of the SU-8 blocks. (**a**) Entire view. (**b**) Yield of released blocks. Microscope images of blocks with designed values of (**c**) 500 μm × 1000 μm, (**d**) 750 μm × 1500 μm, (**e**) 1000 μm × 2000 μm, and (**f**) 1250 μm × 2500 μm (Width × Length). (**g**) Widths and (**h**) lengths of SU-8 blocks versus designed values.

Four patterns of submillimeter-sized SU-8 blocks were fabricated (Figure 7) and released from the wafer after dissolution of a dextran layer. The thickness of the blocks was 282.9 ± 23.9 μm (N = 20). This block thickness fits the height of the PDMS channel. Figure 7a shows SU-8 blocks on a dextran layer and Figure 7b gives the yield of released blocks. The major reason of lowering the yield is the insufficient formation of a sacrificial layer on a wafer. This yield can be improved with uniform coating of dextran by surface treatment before spin-coating. Figure 7c–f present microscope images of each block. The measurement results of the block width and length are plotted in Figure 7g,h. The length and width of blocks were almost the same as the design values.

### 3.3. Unidirectional Transport of a Block by V. carteri

**Figure 8:**
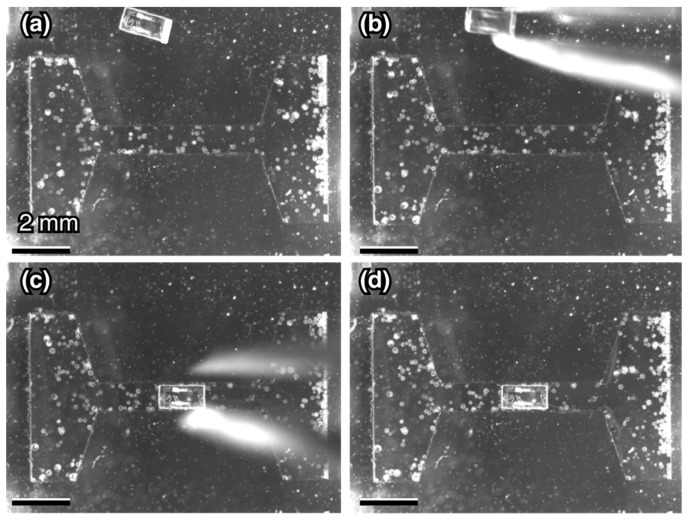
Placement of a 750 μm width block in a 970 μm width micro channel with tweezers. (**a**) Initial position of the block. (**b**) Pickup of the block. (**c**) Positioning of the block at the center of the channel. (**d**) Final position of the block.

The suspension of *V. carteri* colonies were injected into an open top chamber. The LEDs on one side were used to control the motion of the colonies and migrated them to the side of the chamber. Figure 8 shows the placement of an SU-8 block in the center of the channel using tweezers, while the colonies migrated to the right. This manual handling did not break the SU-8 blocks. Since SU-8 has higher density [47] than water, the block sedimented in water and were in a stationary state. We confirmed a drift flow was negligible in an open top chamber. A chamber was in a steady state 3 s after a disturbance was manually exerted on the chamber.

**Figure 9:**
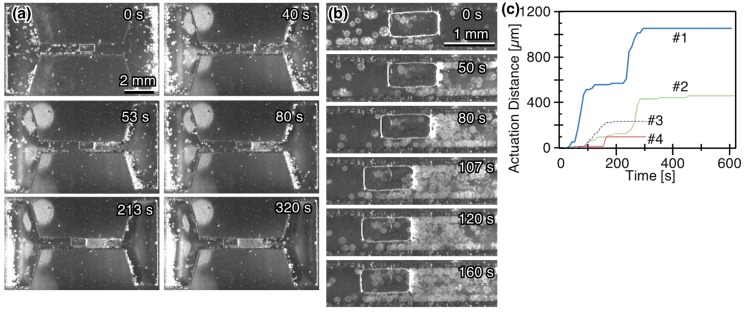
Unidirectional transport of a block in a microchannel. Time series micrographs showing the actuation results of a block in (**a**) the whole chamber and (**b**) the central region. A 500 μm width block was put in a 710 μm width channel. (**c**) Travel distances of the actuated blocks by *V. carteri*. The data were labeled as #1–4 based on the travel length order. #1–3 and #4 were taken with a 500 μm and 750 μm width blocks in a 710 μm and 970 μm width channel, respectively.

We demonstrated the unidirectional transportation of a block in a guide channel using phototaxis in *Volvox* (Figure 9). 3 of the 5 trials with a 500-μm width block and 1 of 5 trials with a 750 μm width block were transported by *V. carteri*. Figure 9a,b and Supplementary Movie S1 show the migration of *V. carteri* to the left irradiation direction and the movement of a block toward the left side. About 10 colonies were adjacent on the right side of the 500 μm width block, around 50 colonies were accumulated behind the adjacent colonies. Even when the almost same number of colonies collided with the block while pushing it, the block was intermittently actuated. About 10 colonies were sandwiched between the block and guide and they are presumed to be obstacles. Without the collision of *Volvox*, a block was not transported. Figure 9c is the travelling lengths of actuated blocks. The experiments were labelled in descending order of travelling lengths. The driving speeds were calculated from the structure driving distance in each of these sections, and the fastest driving speed was 24 μm/s at around 240 s in experiment #1. The adhesion of the block to the channel was prevented by coating them with Pluronic. Compared with the rotation and translation of a gear by *V. aureus* [32], we achieved the unidirectional transport of a cargo without rotation. This restricted motion was enabled by the combination use of *Volvox*, a guide structure, cargo, and light irradiation platform. 

As the large variations in the travel distances and the step-like pattern were displayed in Figure 9c, the possible reasons are discussed. We consider resistance and driving forces on a block and characterize the dynamics of the transportation. The drag forces on a block are the viscous forces against the block and the friction forces between the block and a guide or *V. carteri*. When a bubble smaller than a block remained between a block and guide, the bubble pinned the block. In such a case, a pinning force should be considered. The driving forces are thrust generated by *V. carteri*. A cargo started to be transported by the *Volvox* colonies, when the total driving forces of *V. carteri* exceeded the total resistances forces on the cargo. On the other hand, a block stopped when the total restriction forces were greater than the total propulsive forces.

*V. carteri* generates a horizontal driving force while *V. carteri* laterally swim. We calculate the viscous force on swimming *Volvox* in the fluid in the lateral direction. A swimming force of *Volvox*, *F*, is calculated considering Stokes’ drag represented by the following formula.

(1) F=6πηRV

where *η* is the viscosity of the medium, *R* is the radius of *V. carteri*, and *V* is swimming speed of *V. carteri*. The medium viscosity at 20 °C is *η* = 1.0 mPa s. The speed is obtained from the previous experiment in Section 3.1, and we assume *V* = 200 μm/s. The average diameter of swimming *Volvox* was 2*R* = 140 ± 20 μm (N = 30). Substituting these values into Equation (1) gives the driving force of *V. carteri*, *F* = 0.26 nN. When 10 colonies push the block in one direction, the total force is simply calculated to be the sum of 10 colonies and in the order of 3 nN. This calculated force is probably overestimated because each *V. carteri* maintained a portion of its thrust when pushing the block and only a portion of the propulsive thrust of *V. carteri* was used to push the block. In practice, the swimming direction of all the colonies is not aligned for the entire time and this variation of the driving forces is considered to cause the intermittent actuation of a block. 

## 4. Conclusions

We picked up a microalgae, *Volvox carteri*, and demonstrated the one-way transportation of a block based on its phototaxis, which allowed for the use of this algae as a microrobot. We evaluated the phototaxis and measured the swimming speeds of *Volvox* colonies in a closed square micro chamber. Approximately 50% of total colonies migrated to the area closest to the lit LED. We prepared a guided structure, cargo, and light irradiation platform for a unidirectional actuation. We established the fabrication process of a thick SU-8 mold and block with diluted SU-8 and the rubber sheet. A sacrificial layer was formed underneath the blocks, and the dextran layer was dissolved to release the blocks. 

All components were integrated and used to achieve the block actuation. The block was intermittently transported by the swimming forces of the *Volvox* colonies. The dynamics of the block actuation was characterized and the restriction and driving forces on the block were calculated to understand the actuation principle. We observed the large variations in the travel distances and the step-like pattern, accordingly, the experimental setup should be improved to remove the effect of the restrictive forces. We hypothesized that *V. carteri* (neutral swimmer) should be adjacent to the wall of a block for actuating a cargo in the swimming direction. Adjacent *V. carteri* colonies transported the cargo unidirectionally by colliding with the cargo. We consider pusher swimmers transport a cargo at a distance from the cargo.

Our developed technologies are fundamental for accomplishing one-way actuation of a submillimeter-size cargo by *Volvox*. As our group reported a microfluidic valve based on the *Volvox* outer wall [48], we envisage higher-level applications in microfluidic systems with the use of positive phototaxis of microorganisms and an extension tool. Integration of this block actuation mechanism into the system will lead to broaden the application fields. Phototactic microrobots enable us to make smart microdevices that can change their functions dynamically by responding to an optical environment. According to the same principles of autonomous microsystems based on stimuli-responsive hydrogels [49], light-sensitive motors will bring autonomous microsystems such as fluidic valves, sorters, regulators, pumps, mixers, drug-delivery devices, fluidic cooling devices, and liquid lenses.

## Supplementary Materials

The following are available online at http://www.mdpi.com/2072-666X/10/2/130/s1, Movie S1: The unidirectional actuation of a block in the whole chamber. This video is eight times faster than real time. The development of the light illumination platform and the fabrication method of a square micro-chamber are provided in the Supplementary data.
