# Supplementary material for: Phototactic Algae-Driven Unidirectional Transport of Submillimeter-Sized Cargo in a Microchannel
Source: Micromachines (Basel). 2019 Feb 16;10(2):130. doi: 10.3390/mi10020130 (PMC6412834; doi:10.3390/mi10020130)
Supplement: Supplementary file 1 [file micromachines-10-00130-s001.zip › Supplementary Materials.docx]

Supplementary Materials

Phototactic Algae-Driven Unidirectional Transport of Submillimeter-sized Cargo in a Microchannel

Moeto Nagai, Takahiro Hirano and Takayuki Shibata

Development of Light Illumination Platform

**Figure S1.** Illumination platform. (a), (b) design views of the outer frame. (c) Constructed irradiation platform.

We designed an out-of-frame for fixing LEDs in Solid Works and developed a light irradiation platform (Figure S1). The outer frame was machined from an aluminum alloy block. This frame held the installed LEDs and surrounded a chamber. The inner space of the outer frame was 18 mm×18 mm. LEDs were inserted in four directions of the frame. The size of a PDMS chamber was 15 mm (vertical) × 15 mm (horizontal) × 0.3 mm (height).

Fabrication Method of a Square Micro-chamber

**Figure S2.** Schematic of microchamber fabrication.

We fabricated a microchamber for the demonstration of phototactic control. The printing plate was treated with a 10 μL (1H,1H,2H,2H-perfluorooctyl) silane (PFOCTS; Sigma-Aldrich, USA) by vaporizing it for 90 s at 60 °C. The silanized resin relief plate was molded in PDMS and peeled from the resin relief plate. PDMS was cured for 24 hours at room temperature. The inlet and outlet of PDMS was punched with a 0.5 mm diameter. The PDMS chip and a glass substrate were treated for 30 s (45 W, 20 kHz, pressure 10–100 Pa) with a vacuum plasma equipment (YHS-R, SAKIGAKE-Semiconductor Co., Ltd, Japan) and bonded together.
